# Improvements in Performance of Nursery Pigs Provided with Supplemental Oil Derived from Black Soldier Fly (*Hermetia illucens*) Larvae

**DOI:** 10.3390/ani12233251

**Published:** 2022-11-23

**Authors:** Eric van Heugten, Gabriela Martinez, Alejandra McComb, Liz Koutsos

**Affiliations:** 1 Department of Animal Science, North Carolina State University, Raleigh, NC 27695-7621, USA; 2 EnviroFlight, LLC, Apex, NC 27539, USA

**Keywords:** black soldier fly larvae, insects, lauric acid, nursery pigs, oil

## Abstract

**Simple Summary:**

Black soldier flies are a non-nuisance insect species, and their larvae can be used to efficiently convert low quality byproduct ingredients into valuable nutrient-rich biomass. The lipid fraction obtained from black soldier fly larvae contains high concentrations of lauric acid, which is highly digestible and has antimicrobial properties. The objective of the present study was to determine if black soldier fly larvae oil could improve the growth and efficiency of newly weaned pigs, which are especially compromised due to the stress associated with the weaning process. We conclude that supplemental black soldier fly larvae oil, replacing equal amounts of corn oil, increased average daily gain and improved feed efficiency, resulting in increased final pig body weight after the 40-day test period

**Abstract:**

The current study evaluated the impact of increasing levels of supplemental black soldier fly larvae (BSFL) oil, a novel and sustainable feed ingredient, on growth performance and blood chemistry indices in nursery pigs. Pigs were weaned at 21 days of age (*n* = 192; body weight = 6.9 ± 0.15 kg) and randomly allotted within sex and body weight to 1 of 4 dietary treatments, using 48 pens (4 pigs/pen). Treatments contained 0, 2, 4, or 6% supplemental BSFL oil, replacing equivalent quantities of corn oil. A 3-phase feeding program was used with 14, 11, and 15 days for phase 1 to 3, respectively. Supplementation of BSFL oil linearly (*p* ≤ 0.052) improved pig body weight and daily gain throughout the study and overall. No differences were observed in feed intake; however, feed efficiency was linearly improved with BSFL oil supplementation for phase 1 and 2 (*p* < 0.05). Serum cholesterol increased linearly (*p* = 0.002) and platelet count tended to increase linearly (*p* = 0.082) with increasing BSFL oil. No other differences were detected in blood chemistry and all results were within normal ranges. In conclusion, BSFL oil is a valuable, energy-dense feed ingredient that can promote growth performance of nursery pigs.

## 1. Introduction

Products derived from insects can provide a sustainable alternative to traditional feed ingredients used in commercial animal production. Indeed, insects can be reared very efficiently on lower quality feed ingredients, yielding high quality protein, lipids, and energy, while at the same time using less land and water resources compared to other plant and animal sources [1,2,3]. Each insect species has its own distinct nutritional composition based on life stage, environmental factors, and diet, all of which can be controlled when rearing insects commercially [2]. The black soldier fly has great potential as an insect species for successful commercial production. They are relatively easy to grow, the adult fly does not eat and is not a vector for disease, the larval stage is very rich in nutrients, and the larvae are able to efficiently consume and convert organic materials into nutrient-rich biomass that can be recycled back into the food chain. Black soldier fly larvae (BSFL) contain approximately 32 to 48% crude protein (non-chitin corrected) and 12 to 42% crude fat on a dry basis, depending on the substrate on which they were reared [4,5,6]. The larvae can be further processed by defatting into a high protein meal (>60% crude protein), also yielding a concentrated lipid source, and the production of other potential products, such as chitin and antimicrobial peptides. The protein fraction of BSFL has a high-quality amino acid profile with high digestibility [7,8,9,10,11] and has been demonstrated to be a valuable protein source in poultry [12], swine [13], and fish [14]. The lipid fraction of BSFL is notably high in saturated fatty acids ranging from 51.7 to 86.9% of total fatty acids, with a mean of 70.1% [15,16]. The main fatty acid present in BSFL oil is lauric acid (C12:0), averaging 42.4% of total fatty acids, with myristic (11.0%), palmitic (13.7%), oleic (13.9%), and linoleic (16.2%) acid representing other fatty acids present at large quantities [15]. It should be noted that the fatty acid composition of BSFL oil can be easily modulated by using different rearing diets to achieve specific targeted fatty acid composition, although lauric acid typically remains present at high concentrations [17,18,19,20].

Relatively few studies have evaluated BSFL oil as a potential alternative to common lipid sources used in various food animal species. In general, no adverse effects were observed on growth performance of broilers, turkey, quail, and rabbits when BSFL oil replaced conventional lipid sources, including coconut oil, corn oil, soybean oil, and palm oil [15]. Black soldier fly larvae oil is an interesting ingredient for use in food animal production because of its high concentration of lauric acid. Medium-chain fatty acids, including lauric acid, have been reported to have immunomodulatory properties and exhibit antimicrobial activity to bacteria and enveloped viruses [21,22]. In addition, medium-chain triglycerides are digested and absorbed more efficiently than long-chain fatty acids and are preferentially oxidized for the production of energy [23]. These properties may be especially relevant in newly weaned pigs. The weaning process in commercial pig production is an abrupt, stressful event, resulting in very low initial feed intake, compromised intestinal health and function, reduced digestion capacity, and diminished immune response.

We hypothesized that BSFL oil can provide a digestible source of lipids and energy to newly weaned pigs and, through its unique fatty acid profile, can improve the growth performance of nursery pigs. Thus, the objective of the present study was to determine the effects of incremental levels of BSFL oil, substituting equivalent quantities of corn oil, on growth performance, serum chemistry, and complete blood count of early weaned nursery pigs.

## 2. Materials and Methods

### 2.1. Experimental Design and Animal Management

A total of 192 crossbred barrows and gilts (Smithfield Premium Genetics, Roanoke Rapids, NC, USA; body weight = 6.9 ± 0.15 kg), weaned at 21 days of age, were randomly allotted within body weight and sex to 1 of 4 dietary treatments, using an Excel based allotment program [24]. Pigs were housed in 48 nursery pens (16 pens in each of 3 near identical nursery rooms) with 4 pigs per pen, resulting in 12 blocks per dietary treatment. For block 1 and 2, each pen had 3 barrows and 1 gilt and for blocks 3 to 12, each pen housed 2 barrows and 2 gilts per pen. Each pen of 4 pigs was subsequently evaluated for the presence of littermates. If littermates were present within a particular pen, one of the littermates was exchanged with a pig with the approximate same body weight and of the same sex from another pen within the same weight block. This process ensured that littermates were distributed between treatments (within block), rather than being located within pen. Treatment groups were randomly allocated to the experimental units (pens).

Nursery pens were 0.91 by 1.52 m in dimension with triangular steel flooring (Tri-Bar, Nooyen Manufacturing Inc., Mt. Sterling, KY, USA). Each pen contained 2 nipple water drinkers in the back of the pens and a double space stainless steel feeder located in the front of the pens (Staco, Inc., Schaefferstown, PA, USA). Lighting consisted of fluorescent lights controlled by a timer. There were six light fixtures on each side of the room, each containing two fluorescent bulbs. Lights were turned on from 6:00 a.m. until 8:00 p.m. and turned off from 8:00 p.m. until 6:00 a.m. Temperatures in the nurseries were set at 28 °C for the first 18 days, followed by a drop in temperature of 1 °C every 2 to 4 days until the temperature setting reached a low of 19 °C. Air was mixed using 2 stir fans per room. Rooms had side wall baffle systems to allow for fresh air inflow and ventilation was maintained to exceed minimum air flow requirements [25]. Manure was managed as a flush system and pits were flushed 2 times per day.

Pigs were given unlimited access to feed and water during the 40-day experimental period. Fresh feed was added to the self-feeders as needed to ensure that fresh feed was always available. Feed consumption was calculated weekly from feed added to the feeder minus feed left in the feeder at the end of the feeding phase minus waste feed. Water was provided on ad libitum basis and waterers were checked two times per day to ensure adequate water flow. Temperature and humidity were checked daily to verify proper environmental conditions were maintained. Pigs were observed twice daily for any possible signs of illness. Pigs which looked unthrifty as evidenced by rough hair coat, sunken belly, diarrhea, or weight loss, or pigs that displayed mobility problems, swollen joints, or other signs of illness were closely observed. When deemed necessary, pigs were treated with antibiotics per the advice of a licensed veterinarian. Incidences of medical intervention were recorded. If pigs did not improve after treatment, they were removed from the study. The reason for removal, prior treatments, body weight, and date of the removal were recorded.

### 2.2. Experimental Diets and Manufacturing

Pigs were fed 1 of 4 dietary treatments, consisting of 0, 2, 4, and 6% supplemental BSFL oil (EnviroFlight LLC, Apex, NC, USA), replacing refined edible-grade corn oil (WebstaurantStore, Lancaster, PA, USA) on a 1:1 basis. This approach assumed that the metabolizable energy (ME) content of BSFL oil was similar to corn oil in diet formulation. Diets were formulated using the National Swine Nutrition Guide formulation software [26]. Diets met or exceeded nutrient recommendations established by the NRC [27] and they were fed in 3 phases throughout the nursery (Table 1). Phase 1 diets were fed from day 0 to 14, phase 2 diets from day 14 to 25, and phase 3 diets from day 25 to 40.

Diets were manufactured at the North Carolina State University Feed Mill Education Unit. Whole corn was ground using a hammer mill (Model 1522, Roskamp Champion, Waterloo, IA, USA) to pass through a 2.2 mm screen (grind size of 600 to 800 microns). To create the experimental diets, a basal mix was manufactured first, containing only dry ingredients. Dry ingredients were blended in a double ribbon mixer (Model TRDB126-0604, Hayes and Stolz, Fort Worth, TX, USA). This mix was then divided into 4 equal size batches and the appropriate levels and type of oil were then added to make the final dietary treatments. For the addition of lipids, a total of 1% oil was added to the meal first in the mixer. Corn oil was used for this purpose for all diets, except for the diet with BSFL oil replacing all the corn oil, in which case 1% of BSFL oil was added to the meal in the mixer. The appropriate remaining oil (5%) was added directly onto the pellets in a 225 kg capacity mixer for phase 1 and 2 diets (which were offered as pelleted diets) and mixed for 30 s. For the pelleting process for phase 1 and 2 diets, diets were conditioned at approximately 74 °C and pellets were produced with a pellet mill (model PM1112-2, California Pellet Mill Co., Crawfordsville, IN, USA) using a 4.4 × 25 mm die. Pellets were cooled with ambient air in a counter-flow cooler (Model VK09x09KL, Geelen Counterflow USA Inc., Orlando, FL, USA). Phase 3 diets were manufactured in meal form. For these diets, lipid sources were supplemented to the basal phase 3 mixture in an 1800 kg capacity mixer to create the final experimental diets.

### 2.3. Sampling and Measurements

Pigs were weighed individually at the start of the study and on day 7, 14, 21, 25, 33, and 40 to calculate average daily gain (ADG), which was then averaged by pen. Feed additions to the feeders were recorded and leftover feed in the feeders was determined at the end of each period at the same time pigs were weighed. Feed disappearance was calculated from feed added to the feeder minus feed left in the feeder minus feed removed due to spoilage. Average daily feed intake (ADFI) per pen was then calculated from feed consumed during the period divided by the total number of days for pigs within each pen. Feed efficiency was calculated as the ratio of average daily gain for each period (or phase) divided by the average daily feed intake for the period.

Blood samples were collected at the end of the nursery period (day 40) from one median weight pig per pen. Blood was collected via jugular venipuncture in plain vacuum tubes (for serum) and tubes with K_3_-EDTA (for whole blood). Serum was collected following centrifugation of blood at 1000× *g* for 20 min at 10 °C. Serum samples were analyzed for total protein, albumin, globulin, aspartate aminotransferase, alanine aminotransferase, alkaline phosphatase, γ-glutamyltranspeptidase, urea N, creatinine, glucose, Ca, P, Mg, K, Na, Cl, cholesterol, triglycerides, amylase, lipase, and creatine phosphokinase. Whole blood samples were analyzed for white blood cells, red blood cells, hemoglobin, hematocrit, platelets, neutrophils, lymphocytes, monocytes, eosinophils, and basophils. Analyses were conducted by the Antech Diagnostics laboratory (Cary, NC, USA) using an auto-analyzer (Olympus AU 5400; Olympus America Inc., Melville, NY, USA).

Subsamples of feed were collected during load-out of the feed from the feed mixer (or pellet cooler) to the bagging unit. Ten subsamples were obtained at a port in the auger line at equally spaced intervals between the beginning of load out and the end of load out. Representative samples of the mixture (1000 g) were obtained by splitting the samples from the mixer using a sample splitting device. Samples for phase 1 diets were analyzed for proximate composition, fiber, minerals, and amino acids. Samples for phase 2 diets were analyzed for proximate composition, fiber, minerals, and comprehensive fatty acid composition and samples for phase 3 diets were analyzed for proximate composition, fiber, and comprehensive fatty acid composition. All feed samples were analyzed by Midwest Laboratories (Omaha, NE, USA).

### 2.4. Statistical Analyses

Data were analyzed as a randomized complete block design by analysis of variance (ANOVA) using the General Linear Models procedure of SAS (SAS Inst. Inc., Cary, NC, USA) to examine the effect of BSFL oil inclusion on dependent variables. Pen served as the experimental unit for performance and pig (one pig per pen) was the experimental unit for blood analysis. Orthogonal linear and quadratic contrast comparisons were made to determine the impact of BSFL oil inclusion at 0, 2, 4, and 6% when replacing corn oil. Proper coefficients for linear and quadratic orthogonal contrasts were calculated using Proc IML. Data were expressed as least squares means and differences were deemed to be statistically significant at *p* ≤ 0.05 and tendencies when 0.05 < *p* ≤ 0.10.

## 3. Results and Discussion

### 3.1. Ingredient and Feed Analyses

Black soldier fly larvae oil in the present study was obtained from 2 different lots. Lot 1 was used in diets fed during phase 1 and 2. Lot number 2 was used to manufacture diets for phase 3. The analyzed fatty acid composition of both lots was very similar, indicating that variation between lots for fatty acid composition was minor (Table 2). Total fat content determined after acid hydrolysis was 99.1 and 99.2% for lot 1 and 2, respectively. Therefore, the BSFL oil was relatively pure with very low moisture, impurities, and unsaponifiable compounds (less than 0.9 and 0.8% for lot 1 and 2, respectively).

Black soldier fly larvae oil contained high concentrations of lauric acid (C12:0), averaging 36.9 g/100 g of lipid for the two lots used in the current study. Other fatty acids that were present at high concentrations were linoleic (C18:2), palmitic (C16:0), oleic (C18:1), and myristic (C14:0) acid at concentrations of 17.3, 14.6, 13.1, and 9.8 g/100 g of lipid, respectively. The composition of the BSFL oil was very similar to the values reported previously [15]. Although the composition of BSFL can be manipulated [17,18,19,20], the conditions of rearing and dietary substrate used in commercial production of BSFL are tightly controlled as part of a quality assurance program with the aim of yielding products with a specific and consistent nutrient profile.

When manufacturing diets, we prepared a basal mix within each phase and divided this basal mix into equal portions to manufacture the final treatment diets. Thus, treatment diets within dietary phase were identical in their ingredient make-up and the nutrient composition of diets should be similar between treatments, with the exception of fatty acid composition. The proximate chemical, fiber, mineral, and amino acid composition of dietary treatments was in accordance with target nutrient values and well within expected laboratory variation. Analyzed fatty acid composition for phase 2 and 3 diets (Table 3) confirmed the proper composition of the experimental diets. phase 2 diets contained 0 (not detectable), 0.63, 1.11, and 1.79 g of lauric acid per 100 g of total fatty acids for 0, 2, 4, and 6% supplemental BSFL oil, respectively, whereas phase 3 diets contained 0.01, 0.66, 1.22, and 1.95 g of lauric acid per 100 g of total fatty acids for 0, 2, 4, and 6% supplemental BSFL oil (Table 3), respectively. This clearly demonstrates incremental increases in concentrations of lauric acid as expected, consistent with the incremental increasing levels of BSFL oil added to the experimental diets.

### 3.2. Growth Performance

Two pigs (0% BSFL oil treatment) were removed from the study due to failure to thrive as indicated by progressive weight loss while on test and 1 pig (4% BSFL oil) was removed due to prolapse. Four pigs (1 pig fed 0% BSFL oil and 2 pigs fed 4% BSFL oil) died during the course of the experiment, representing a death loss of 2.1%. A summary of productivity records of the pork industry showed a mortality of 3.62% in nursery pigs for commercial farms in the top 25th percentile (mean was 4.58%) [28]. Some death losses are expected and death losses in university facilities with low stocking density and good health are expected to be modest. None of the pigs in the current study were medically treated with injectable medication or oral antibiotics.

In general, pigs in the present study performed very well as indicated by a mean ADG of 532 g/d, gain:feed ratio of 762 g/kg, and a final body weight of 28.2 kg, reached in 40 days in the nursery (Table 4). This compares to a mean ADG of 444 g/d, a G:F ratio of 671 g/kg, and a final BW of 26.9 kg (after 46 days in the nursery) for the top 25th percentile of a large sample group of pork production companies [28]. Supplementation of BSFL oil, replacing corn oil, generally improved growth performance of nursery pigs. Pig body weight linearly increased with increasing BSFL oil in the diet on day 14 (*p* = 0.018), day 21 (*p* = 0.010), day 25 (*p* = 0.014), day 33 (*p* = 0.021), and at the end of the study (*p* = 0.052). This increase in body weight appeared to be due primarily to increased ADG early in the study as evidenced by increased ADG during day 7 to 14 (*p* = 0.002), phase 1 (day 0 to 14; *p* = 0.017), and phase 2 (day 14 to 25; *p* = 0.055), resulting in improved overall ADG (*p* = 0.048). No significant differences in average ADFI were observed due to BSFL oil inclusion, implying that BSFL oil is a palatable ingredient for nursery pigs. This agrees with studies feeding whole, live BSFL to weaned pigs showing high palatability of BSFL [29,30]. Improvements in ADG without changes in ADFI resulted in improved gain:feed ratio (feed efficiency) during day 7 to 14 (*p* < 0.001), day 14 to 21 (*p* = 0.054), phase 1 (day 0 to 14; *p* < 0.001), and phase 2 (day 14 to 25; *p* = 0.049).

The impact of feeding BSFL oil in pigs has not been previously published. In other species, potential effects of BSFL oil have been reported, showing no benefits when fed to broilers, turkey, quail, and rabbits when BSFL oil partially or totally replaced conventional lipid sources, including coconut oil, corn oil, soybean oil, and palm oil [15]. Recent studies confirm these observations. In laying hens, substitution of 50 or 100% of soybean oil with cold-pressed BSFL oil did not impact productive performance [31]. In a similar study Kieronczyk et al., (2022) reported no effects of cold-pressed BSFL oil replacing 50 or 100% of soybean oil on growth performance of young turkeys [32]. Although no peer-reviewed publications reporting effects of BSFL oil in pigs could be found, studies using full-fat BSFL in diets for pigs may provide some insight into potential impacts of oil derived from BSFL. In nursery pigs, feeding of 1, 2, or 4% full-fat BSFL meal in replacement of fishmeal and soybean oil (up to 1.15%) linearly improved ADG and feed efficiency [33]. In another study [34], no beneficial effects were noted on nursery pig performance when full-fat BSFL were included at up to 19.1%, replacing soybean meal, soy protein concentrate, fish meal, and rapeseed oil (BSFL meal contributed up to 69% of the total dietary fat). The digestible energy (DE) and ME contents in full-fat and defatted BSFL have been shown to be high, with full-fat BSFL providing more DE (4.93 Mcal/kg) and ME (4.57 Mcal/kg) than defatted BSFL (3.94 and 3.40 Mcal/kg for DE and ME, respectively) [11]. Clearly, impacts of additional oil from full-fat BSFL meal on pig performance cannot be distinguished from the potential impact of the protein or chitin fraction of BSFL meal. Collectively, reported data generally show no clear positive effects of BSFL oil on productive performance, which is in contrast with results reported in the present study.

Growth rate of pigs fed BSFL oil in the current study was improved by 18.1% during phase 1, and 5.6% during phase 2, resulting in improved overall ADG and increased body weight at the end of the nursery period. The improved ADG immediately after weaning is especially noteworthy considering that this is an important transition period that correlates with subsequent growth [35,36]. The performance benefits observed in the current study may be related, in part, to the high concentration of lauric acid in BSFL oil. Lauric acid is amongst the medium-chain fatty acids that are generally defined as having chain lengths of 6 to 12 carbon atoms.

Lauric acid has been reported to exhibit antimicrobial activity to bacteria, especially Gram-positive bacteria, and enveloped viruses and has demonstrated immunomodulatory properties [21,22,23]. Medium-chain triglycerides are digested and absorbed more efficiently than long-chain triglycerides and are transported directly to the liver via the portal vein for preferential β-oxidation to produce energy [23]. The rapid absorption of medium-chain fatty acids may preclude sufficient quantities to be present in the gastrointestinal tract to exert antimicrobial effects [37,38,39]. Thus, the use of medium-chain fatty acids bound as part of triglycerides, rather than free fatty acids, may be beneficial, although this ultimately depends on sufficient endogenous lipase activity for the hydrolysis of triglycerides [37,38].

### 3.3. Serological and Hematological Indices

Serum chemistry and complete blood count data were collected to verify normal functioning and health of pigs (Table 5 and Table 6). All values were within expected and acceptable ranges [40] and were consistent with our previously published results in growing pigs [41]. Supplementation of BSFL oil during the 40-day experimental period did not significantly alter serum chemistry or complete blood counts, which agrees with studies in poultry [32,42,43]. The only exception was cholesterol. Serum cholesterol concentrations linearly (*p* = 0.002) increased with increasing BSFL oil in the diet. Cholesterol is naturally present in BSFL oil, whereas corn oil does not contain cholesterol, and may have directly contributed to increased serum cholesterol concentrations. Harris et al., (2003) reported increased plasma cholesterol and low-density lipoprotein cholesterol concentrations in pigs fed tallow or coconut oil compared to corn oil; however, no differences were detected in muscle [44]. In young turkeys, partial or total replacement of soybean oil by BSFL oil increased serum concentrations of cholesterol and low-density lipoprotein cholesterol but did not affect cholesterol concentrations in breast meat or leg meat of turkeys [45]. On the other hand, no differences in serum cholesterol concentrations were detected when soybean oil was partially or totally replaced with BSFL oil in the diets of broilers [32,42,43]. Kim et al., (2022) observed no differences in cholesterol concentration of eggs from hens supplemented with BSFL oil in replacement of soybean oil [31]. The serum cholesterol concentrations reported in the current experiment were within expected published ranges for nursery pigs [40] and are unlikely to impact cholesterol concentrations in final edible products.

## 4. Conclusions

Results of the present study indicated that increasing supplemental BSFL oil from 0 to 6% in increments of 2% increased average daily gain and improved feed efficiency of nursery pigs in a linear manner, especially early after weaning, resulting in increased final body weight after the 40-day experimental period. Serum chemistry and total blood count were within normal ranges for young pigs suggesting that health status was not impacted by BSFL oil. Serum cholesterol linearly increased with BSFL oil supplementation, which may be related to the fact that BSFL oil contains some cholesterol, whereas corn oil does not, and BSFL oil contains cholesterogenic fatty acids (similar to coconut oil and palm kernel oil). In conclusion, BSFL oil is a valuable, energy-dense feed ingredient that can be successfully fed to nursery pigs, while improving their growth performance.

## Figures and Tables

**Table 1:** Composition of experimental control diets fed during nursery phase 1 (day 0 to 14), 2 (day 14 to 25), and 3 (day 25 to 40).

Ingredient	Diet Phase		
	1	2	3
Corn, yellow dent	31.38	48.35	55.36
Soybean meal, 47.5% CP	24.50	29.80	34.66
Whey, permeate	22.50	6.25	-
Poultry byproduct meal	8.00	4.00	-
Plasma, spray-dried	5.00	2.00	-
L-lysine·HCl (78.8% lysine)	0.261	0.340	0.361
DL-methionine	0.210	0.194	0.179
L-threonine	0.115	0.137	0.140
L-tryptophan	-	0.004	-
Monocalcium phosphate, 21% P	0.660	1.264	1.534
Limestone	0.629	0.863	1.156
Salt	0.150	0.350	0.400
Copper sulfate, 25.2% Cu	0.063	0.053	-
Zinc oxide, 72% Zn	0.324	0.185	-
Vitamin premix ^1^	0.055	0.055	0.055
Mineral premix ^2^	0.150	0.150	0.150
Corn oil ^3^	6.00	6.00	6.00
**Calculated Composition**			
Metabolizable energy, Mcal/kg	3.59	3.57	3.56
Net energy, Mcal/kg	2.65	2.68	2.68
Lactose	18.0	5.0	0
Crude protein, %	24.61	23.11	21.66
Crude fat, %	8.76	8.76	8.45
Ca, %	0.85	0.80	0.80
Total P, %	0.77	0.75	0.72
Available P, %	0.50	0.45	0.40
Standardized ileal digestible amino acids, %			
Lys	1.50	1.40	1.30
Thr	0.93	0.87	0.81
Met	0.51	0.49	0.47
Met+Cys	0.87	0.81	0.75
Trp	0.27	0.25	0.23
Val	0.99	0.91	0.85

^1^ Supplied the following amounts per kilogram of complete diet: 11,313 IU of vitamin A, 1613 IU of vitamin D_3_ as activated animal sterol, 65 IU of vitamin E as dl-α-tocopheryl acetate, 5.3 mg of vitamin K as menadione dimethylpyrimidinol bisulfate, 8.1 mg of riboflavin, 48.5 mg of niacin, 32.3 mg of d-pantothenic acid as calcium pantothenate, 2.4 mg of folic acid, 0.33 mg of d-biotin, and 0.044 mg of vitamin B_12_. ^2^ Supplied the following amounts per kilogram of complete diet: 16.5 mg of Cu as CuSO_4_, 0.3 mg I as ethylenediamine dihydriodide, 165 mg of Fe as FeSO_4_, 40 mg of Mn as MnSO_4_, 0.3 mg of Se as Na_2_SeO_3_, and 165 mg of Zn as ZnSO_4_. ^3^ Corn oil in the control diet was replaced on a 1:1 basis with 2, 4, or 6% black soldier fly larvae oil to create experimental diets.

**Table 2:** Analyzed fatty acid composition of black soldier fly larvae oil. As fed basis.

Nutrient	Lot Number ^1^	
	1	2
Fat (acid hydrolysis), %	99.1	99.2
Fatty acid concentration, g/100 g		
Lauric (C12:0)	37.3	36.5
Myristic (C14:0)	9.68	10.00
Palmitic (C16:0)	14.8	14.4
Palmitoleic (C16:1)	1.71	2.03
Stearic (C18:0)	2.07	2.24
Oleic (C18:1)	12.6	13.5
Linoleic (C18:2)	17.4	17.1
α-Linolenic (C18:3)	1.52	1.46
Others ^2^	2.02	1.94
Saturated fatty acids	65.1	64.3
Polyunsaturated fatty acids	19.0	18.6
Monounsaturated fatty acids	14.6	15.8
Trans fatty acids	0.40	0.47
Omega 3 fatty acids	1.52	1.46
Omega 6 fatty acids	17.4	17.1
Omega 9 fatty acids	12.6	13.5

^1^ Lot number 1 was used for phase 1 and 2 diets and lot number 2 was used for phase 3 diets. ^2^ Other fatty acids analyzed were present at concentrations of less than 0.75% and consisted of: butyric (C4:0), caproic (C6:0), caprylic (C8:0), capric (C10:0), tridecanoic (C13:0), myristoledic (C14:1 trans), myristoleic (C14:1 cis), pentadecanoic (C15:0), almitelaidic (C16:1 trans), heptadecanoic (C17:0), 10-heptadecanoic (C17:1), eliadic (C18:1 trans), linolelaidic (C18:2 trans), γ-linolenic (C18:3 γ), nonadecanoic (C19:0), arachidic (C20:0), 11-eicosenoic (C20:1), 11-14 eicosadienoic (C20:2), homo- γ linolenic (C20:3), 11-14-17 eicosatrienoic (C20:3), arachidonic (C20:4), eicosapentaenoic (C20:5), heneicosanoic (C21:0), behenic (C22:0), erucic (C22:1), docosadienoic (C22:2), docosapentaenoic (C22:5), docosahexaenoic (C22:6), tricosanoic (C23:0), lignoceric (C24:0), and nervonic (C24:1) acid.

**Table 3:** Analyzed fatty acid composition (g/100 g of diet) of experimental phase 2 (day 14 to 25), and phase 3 (day 25 to 40) diets. As fed basis ^1^.

Fatty Acid	% Black Soldier Fly Larvae Oil Replacing Corn Oil			
	0	2	4	6
Phase 2 diets				
Lauric (C12:0)	n.d. ^2^	0.63	1.11	1.79
Myristic (C14:0)	n.d.	0.18	0.30	0.50
Palmitic (C16:0)	0.96	1.12	1.06	1.18
Palmitoleic (C16:1)	0.04	0.08	0.10	0.14
Stearic (C18:0)	0.18	0.21	0.20	0.22
Oleic (C18:1)	2.16	2.11	1.64	1.50
Linoleic (C18:2)	3.90	3.66	2.74	2.32
α-Linolenic (C18:3)	0.12	0.13	0.14	0.15
Other ^3^	0.14	0.15	0.14	0.18
Phase 3 diets				
Lauric (C12:0)	0.01	0.66	1.22	1.95
Myristic (C14:0)	n.d.	0.18	0.32	0.52
Palmitic (C16:0)	0.91	0.99	0.95	1.10
Palmitoleic (C16:1)	0.02	0.04	0.07	0.10
Stearic (C18:0)	0.15	0.16	0.15	0.18
Oleic (C18:1)	2.10	1.93	1.50	1.32
Linoleic (C18:2)	3.87	3.49	2.58	2.20
α-Linolenic (C18:3)	0.13	0.15	0.14	0.17
Other ^3^	0.12	0.15	0.14	0.15

^1^ Only phase 2 and phase 3 diets were analyzed for fatty acid composition. ^2^ n.d. = not detected. ^3^ Other fatty acids analyzed were present at concentrations of less than 0.05% and consisted of: butyric (C4:0), caproic (C6:0), caprylic (C8:0), capric (C10:0), tridecanoic (C13:0), myristoledic (C14:1 trans), myristoleic (C14:1 cis), pentadecanoic (C15:0), almitelaidic (C16:1 trans), heptadecanoic (C17:0), 10-heptadecanoic (C17:1), eliadic (C18:1 trans), linolelaidic (C18:2 trans), γ-linolenic (C18:3 γ), nonadecanoic (C19:0), arachidic (C20:0), 11-eicosenoic (C20:1), 11-14 eicosadienoic (C20:2), homo- γ linolenic (C20:3), 11-14-17 eicosatrienoic (C20:3), arachidonic (C20:4), eicosapentaenoic (C20:5), heneicosanoic (C21:0), behenic (C22:0), erucic (C22:1), docosadienoic (C22:2), docosapentaenoic (C22:5), docosahexaenoic (C22:6), tricosanoic (C23:0), lignoceric (C24:0), and nervonic (C24:1) acid.

**Table 4:** Growth performance of nursery pigs fed diets with increasing levels of black soldier fly larvae (BSFL) oil replacing equal amounts of corn oil ^1^.

Variable	BSFL Oil Inclusion, %				SEM	*p* Value ^2^	
	0	2	4	6		Treatment	Linear
Body weight, kg							
Initial	6.91	6.90	6.90	6.91	0.009	0.834	0.561
Day 7	8.35	8.42	8.46	8.36	0.087	0.796	0.867
Day 14	9.92	10.07	10.44	10.46	0.179	0.099	0.018
Day 21	13.40	13.59	14.15	13.99	0.190	0.031	0.010
Day 25	16.22	16.40	16.90	16.91	0.223	0.075	0.014
Day 33	21.49	21.26	22.22	22.12	0.262	0.036	0.021
Day 40	27.83	27.62	28.84	28.44	0.340	0.060	0.052
Average daily gain, g/d							
Day 0 to 7	206	217	222	209	12.5	0.783	0.820
Day 7 to 14	224	236	282	300	18.5	0.018	0.002
Day 14 to 21	473	503	530	503	19.2	0.228	0.173
Day 21 to 25	677	703	688	732	23.4	0.393	0.163
Day 25 to 33	659	607	658	650	14.9	0.063	0.714
Day 33 to 40	907	908	942	904	22.4	0.589	0.792
Phase 1 (day 0 to 14)	215	226	252	254	12.8	0.098	0.017
Phase 2 (day 14 to 25)	555	576	588	586	12.0	0.207	0.055
Phase 3 (day 25 to 40)	774	748	795	769	15.0	0.188	0.657
Overall (day 0 to 40)	523	518	548	539	8.5	0.059	0.048
Average daily feed intake, g/d							
Day 0 to 7	215	219	226	211	8.6	0.664	0.956
Day 7 to 14	344	358	364	357	14.0	0.773	0.478
Day 14 to 21	598	571	599	573	15.6	0.434	0.501
Day 21 to 25	851	855	881	855	16.4	0.540	0.609
Day 25 to 33	970	933	995	960	16.8	0.093	0.687
Day 33 to 40	1264	1245	1313	1250	29.1	0.341	0.839
Phase 1 (day 0 to 14)	279	288	295	284	10.1	0.736	0.644
Phase 2 (day 14 to 25)	689	675	701	675	12.7	0.406	0.804
Phase 3 (day 25 to 40)	1107	1079	1140	1095	20.4	0.197	0.780
Overall (day 0 to 40)	690	691	719	696	11.4	0.253	0.365
Gain:feed ratio, g/kg							
Day 0 to 7	944	971	974	981	32.2	0.854	0.432
Day 7 to 14	640	655	762	847	38.7	0.002	<0.001
Day 14 to 21	794	882	889	877	28.7	0.086	0.054
Day 21 to 25	792	820	780	854	24.7	0.171	0.195
Day 25 to 33	681	651	663	678	11.4	0.244	0.938
Day 33 to 40	719	729	715	723	13.6	0.908	0.992
Phase 1 (day 0 to 14)	757	781	844	895	28.3	0.007	<0.001
Phase 2 (day 14 to 25)	808	854	838	866	17.4	0.120	0.049
Phase 3 (day 25 to 40)	701	693	698	703	8.8	0.876	0.814
Overall (day 0 to 40)	759	750	764	774	10.4	0.434	0.208

^1^ Values are least square means of 12 pens per treatment with 4 pigs per pen (total of 48 pens and 192 pigs). ^2^ Quadratic contrast comparisons were not significant for any of the measurements.

**Table 5:** Effects of black soldier fly larvae (BSFL) oil replacing equal amounts of corn oil on serum chemistry in nursery pigs ^1^.

Variable ^2^	BSFL Oil				SEM	*p* Value ^3^	
	0	2	4	6		Treatment	Linear
Total protein, g/dL	5.21	5.37	5.38	5.48	0.112	0.412	0.111
Albumin, g/dL	3.19	3.33	3.33	3.32	0.063	0.357	0.186
Globulin, g/dL	2.02	2.04	2.05	2.16	0.104	0.782	0.361
Albumin:Globulin	1.61	1.65	1.69	1.60	0.081	0.847	0.964
AST, IU/L	30.4	29.9	26.0	27.1	2.63	0.577	0.245
ALT, IU/L	24.7	23.6	22.8	24.0	1.38	0.800	0.650
Alk phosphatase, IU/L	216	227	230	220	18.4	0.945	0.839
GGTP, IU/L	21.5	21.8	23.2	26.2	1.74	0.226	0.055
Urea N, mg/dL	10.8	8.8	10.4	9.3	0.96	0.421	0.467
Creatinine, mg/dL	0.783	0.733	0.767	0.817	0.027	0.188	0.272
Urea N:creatinine	14.1	11.9	13.5	11.6	1.15	0.363	0.260
Calcium, mg/dL	0.71	10.95	10.90	10.88	0.135	0.613	0.436
Phosphorus, mg/dL	10.32	11.00	10.46	10.79	0.238	0.184	0.412
Glucose, mg/dL	117.1	127.0	116.8	124.2	4.42	0.275	0.582
Mg, mEq/L	1.74	1.77	1.70	1.79	0.051	0.620	0.715
Na, mEq/L	142.3	143.0	141.8	141.4	0.63	0.322	0.190
K, mEq/L	6.32	6.59	5.93	6.18	0.279	0.408	0.388
Na:K	23.03	22.26	24.28	23.43	0.919	0.485	0.443
Cl, mEq/L	98.08	98.75	98.33	98.42	0.510	0.831	0.800
Cholesterol, mg/dL	78.4	87.6	92.0	91.0	2.73	0.005	0.002
Triglycerides, mg/dL	71.8	48.4	58.1	57.4	10.10	0.445	0.463
Amylase, IU/L	1190	1287	1244	1294	64	0.641	0.350
CPK, IU/L	3714	3246	3118	2565	950	0.863	0.406

^1^ Values are least square means of 12 pigs (1 median weight pig per pen) per treatment (total of 48 pigs). ^2^ AST = Aspartate aminotransferase; ALT = Alanine aminotransferase; Alk phosphatase = Alkaline phosphatase; GGTP = γ-Glutamyl transpeptidase; CPK = Creatine phosphokinase. ^3^ Quadratic contrast comparisons were not significant.

**Table 6:** Effects of black soldier fly larvae (BSFL) oil replacing equal amounts of corn oil on complete blood counts in nursery pigs ^1^.

Variable ^2^	BSFL Oil				SEM	*p* Value ^3^	
	0	2	4	6		Treatment	Linear
WBC, count × 10^3^/μL	19.9	21.4	19.3	22.1	1.54	0.539	0.507
RBC, count × 10^6^/μL	6.77	6.77	6.58	6.98	0.21	0.643	0.646
Hemoglobin, g/dL	12.00	12.38	12.08	12.74	0.39	0.541	0.281
Hematocrit, %	42.1	42.8	41.6	44.5	1.47	0.531	0.362
MCV, fL	62.6	63.3	62.9	63.6	0.63	0.711	0.353
MCH, pg	17.84	18.30	18.34	18.23	0.18	0.200	0.416
MCHC, g/dL	28.67	29.25	29.25	28.75	0.24	0.173	0.815
Platelets, count × 10^3^/μL	267	413	362	388	38.9	0.063	0.082
Neutrophils, count/μL	8099	7965	7677	8796	959	0.864	0.677
Lymphocytes, count/μL	10,125	11,802	9934	11,379	862	0.347	0.626
Monocytes, count/μL	1187	1235	1132	1463	147	0.416	0.279
Eosinophils, count/μL	373	365	475	462	124	0.885	0.503

^1^ Values are least square means of 12 pigs (1 median weight pig per pen) per treatment (total of 48 pigs). ^2^ WBC = White blood cell count; RBC = Red blood cell count; MCV = Mean corpuscular volume; MCH = Mean corpuscular hemoglobin; MCHC = Mean corpuscular hemoglobin concentration. ^3^ Quadratic contrast comparisons were not significant.

## Data Availability

Data associated with the current study are available from the corresponding author upon reasonable request.
